# Metabolomics Reveals the Response Mechanisms of Potato Tubers to Light Exposure and Wounding during Storage and Cooking Processes

**DOI:** 10.3390/foods13020308

**Published:** 2024-01-18

**Authors:** Xin Wang, Shuiyan Yang, Jinghan Sun, Guoyan Chen, Yunman Wen, Jin Yang, Xuheng Nie, Chao Liu

**Affiliations:** 1 Yunnan Provincial Academy of Food and Oil Sciences, Kunming 650033, China; 2 College of Biological Resource and Food Engineering, Yunnan Engineering Research Center of Fruit Wine, Qujing Normal University, Qujing 655011, China

**Keywords:** potato tuber, light exposure, wounding, metabolomics, steroidal glycoalkaloid, cooking process

## Abstract

Potato is susceptible to light exposure and wounding during harvesting and transportation. However, the metabolite profile changes in these potato tubers are unclear. The potato cultivars in this study included Hezuo88 (HZ88), Shida6 (SD6), and Jianchuanhong (JCH); the effects of light exposure (L), wounding (W), and the cooking process on potato metabolites were determined. In total, 973 metabolites were identified, with differential metabolites (mainly alkaloids, flavonoids, and phenolic acids) accumulated on days 0 and 2 (0 d and 2 d) in the 0dHZ88 vs. 0dJCH (189), 0dHZ88 vs. 0dSD6 (147), 0dSD6 vs. 0dJCH (91), 0dJCH vs. 2dIJCH (151), 0dJCH vs. 2dWDJCH (250), 0dJCH vs. 2dWLJCH (255), 2dIJCH vs. 2dWDJCH (234), and 2dIJCH vs. 2dWLJCH (292) groups. The flavonoid content in the light exposure group was higher than that in the dark group. The alkaloid content in the wounded group was higher than that in the uninjured potato tuber group, but the lipid content in the wounded group was lower. Importantly, only 5.54% of the metabolites changed after potato tuber steaming. These results provide valuable information for the breeding and consumption of potato tubers.

## 1. Introduction

Potato tubers have become one of the four main food crops worldwide from the perspective of yield, they are the main food crop produced in most countries and regions, and they are eaten as a staple food by 1.1 billion people worldwide. Potato tuber accounts for 2 percent of the global energy intake, according to the FAOSTAT (http://www.fao.org/faostat/ (accessed on 5 April 2022)), and it plays significant roles in rural revitalization in China and food security worldwide. A total of 370,436,581 tons of potato tubers were produced worldwide in 2019, with China producing 91,881,397 tons of potato tubers and accounting for 24.8% of the global potato production, according to the FAOSTAT. Chinese potato production and consumption rank first worldwide.

Potato nutrients include carbohydrates, proteins, vitamin C, vitamin B, magnesium, potassium, fiber, and so on [1]. In addition, potato tubers contain some phytochemicals, including anthocyanins, carotenoids, and steroidal glycoalkaloids (SGAs) [2,3,4]. These compounds are beneficial to plants’ resistance to environmental biotic and abiotic stresses [5]. Phenolic compounds protect plants from UV damage by acting as a UV shield [6]. Additionally, potato tubers have many health benefits, including antioxidant and anti-tumor properties, the ability to alleviate diabetes and cardiovascular disease, etc. [7,8]. Therefore, potato and its products have received increasing interest from consumers and researchers.

The cumulative research results show that potato tuber metabolomics are affected by many environmental variables. When potato tubers were stressed by hot climates and mustard (*Sinapis alba* L.), the contents of the phytochemicals were shown to change [9,10]. Noticeably, researchers and consumers should pay attention to the effects of light exposure and wounding on the quality of potato tubers. Potato tubers are susceptible to wounding during harvesting and transportation, which not only causes a certain economic loss, but also rapidly accumulates SGA levels at the site of wounding [11,12]. During storage, the SGA level was shown to increase, especially in the wounded parts of the potatoes. In our previous study, we found that the SGA content changed significantly over time only in approximately 3 mm of flesh at the wounded site. Interestingly, light exposure and wounding had a coupling effect on the variation in the SGA content during potato tuber storage [12], and this change showed a logistic regression pattern [13]. However, the SGA metabolism in potato tubers is different between germinated and dormant potato tubers [14]. These results may be due to the increased cellular metabolic activity in these areas, and researchers have indirectly verified the correlation between cell metabolic activity and SGA content from the perspective of cell substrates and related metabolic enzymes [13]. However, to our knowledge, few studies have investigated potato tuber metabolomics during storage under light exposure and wounding conditions, which should provide insight into these changes.

Researchers have determined the SGA, phenolic, flavonoid, inorganic, and organic contents in potato peel from three commercial potato tubers, and the results showed that there was a wide variation in these compound contents [7]. The influences of cultivar and market class on the potato tuber metabolite profiles were significantly different [15]. The potato phenotypes, such as the potato type and flesh color, were significantly different, so it could be speculated that potato tubers have different metabolites, especially secondary metabolites. Therefore, we selected three representative potato tubers as research objects for widely targeted and targeted metabolomics analyses. To further analyze whether the cooking process has an impact on the beneficial compounds in potato tubers, we conducted a study on the effect of the cooking process on the carotenoid, anthocyanin, and flavonoid contents via targeted metabolomics.

Our research aims to gain insight into the following profiles: (1) the metabolism of different potato cultivars; (2) the metabolic variation affected by light exposure and wounding in potato tubers; and (3) the effect of steaming on potato metabolites. We hypothesized that (1) there were variable metabolomics profiles in the potato cultivars; (2) potato tuber metabolomics affected by light exposure and wounding had significant differences during potato storage; and (3) steaming can reduce the secondary metabolic contents in potato tubers.

## 2. Materials and Methods

### 2.1. Chemicals and Reagents

Anthocyanin standards were supplied by IsoReag (Shanghai, China). The carotenoid standards and formic acid were obtained from Sigma-Aldrich (St. Louis, MO, USA) and BOC (New York, NY, USA), respectively. α-Chaconine and α-solanine references were obtained from Extrasynthese (Genay, France). Hydrochloric acid was acquired from Xinyang Chemical Reagent (Xinyang, China). HPLC-grade methanol (MeOH), ethanol (EtOH), and acetonitrile (ACN) were obtained from Merck (Darmstadt, Germany). BHT was provided by Aladdin. Acetone was supplied by Sinopharm. Methyl tert-butyl ether (MTBE) was supplied by CNW. NaCl was supplied by Rhawn. KOH was supplied by Hushi.

Anthocyanin standards were prepared in 50% MeOH at a concentration of 1 mg/mL, while flavonoid standards were prepared in 70% MeOH at a concentration of 10 mmol/L. Carotenoid standards were prepared in MTBE/MeOH at a concentration of 1 mg/mL, and α-chaconine and α-solanine standards were prepared in MeOH. All of these stock solutions were stored at −20 °C.

Anthocyanin stock solutions were weakened by 50% MeOH to generate working solutions for analysis. Flavonoid stock solutions were diluted with 70% MeOH to produce working solutions prior to examination. SGA (α-chaconine and α-solanine) standards’ working solutions were prepared in line with our established method [16].

### 2.2. Botanical Materials and Scientific Methodology

The potato cultivars in this study included Hezuo88 (HZ88), Shida6 (SD6), and Jianchuanhong (JCH), and samples were obtained from the Yunnan potato market. The experimental design followed that of our previous study [12]. A 150 kg sample was selected for this study, with similar weights for each cultivar (HZ88: 150 ± 30 g; SD6: 200 ± 30 g; JCH: 200 ± 30 g), and the samples did not exhibit visible greening or damage. The potato bulbs were initially held in a fridge at a temperature of roughly 8 °C. Before the test, the potato bulbs were kept in bulk in natural indoor conditions at a temperature of 10~15 °C and a humidity of approximately 62% for 2 weeks for balance, which was regarded as “Day 0”, and then randomly divided into two portions for each bulb. One group of samples was treated with light exposure (about 11 h daily) or dark storage. The other group of samples was treated with injury (cut injury). Specifically, the whole bulbs were sliced into two longitudinal disks at the center, and then the wounds were covered with a 0.1 × 9 × 35 mm white preservative film of polyethylene (PE) (Wenzhou Jiajia Packing Co., Ltd., Wenzhou, China) to moderate moisture release and oxidation, with half of the samples being treated with dark storage and the other half undergoing light exposure treatment (about 11 h daily). These bulbs were stored in bulk for 0, 1, 2, 6, 10, and 20 days at a temperature of ~20 °C and a humidity of approximately 62%. Approximately 5 g of potato samples were employed for wide-target analysis and the precise determination of SGA content (α-chaconine and α-solanine).

Approximately 2 kg of JCH potato was selected and peeled at 0 d, and each tuber was evenly and longitudinally cut in half. These samples were randomly separated into two classes. One class was subjected to steaming, and the other class served as the control (CK). We added 2 L of pure water to the steam pot and steamed potato tubers at 95 °C by placing them above the water for 40 min under a heating power of 1100 W. For the CK, we added 2 L of pure water into another container and placed the potato tubers in the container as described above. Approximately 5 g of samples was collected, and the procedure was executed six times. The samples were placed in plastic bags and promptly preserved in a freezer at a temperature of −20 °C. These samples were used for the accurate determination of anthocyanin, flavonoid, and carotenoid contents.

### 2.3. Sample Preparation and Extraction for Widely Targeted Metabolomics

The samples of potato were subjected to freeze-drying using a vacuum freeze-dryer (Scientz-100F, Ningbo Scientz Biotechnology Co., Ltd., Ningbo, China) for a duration of 48 h, after which they were crushed by employing a mixer mill (MM 400, Retsch, Haan, Germany) with the aid of zirconia beads for 1.5 min at a frequency of 30 Hz. A quantity of 100 mg of potato powder was extracted using a 70% methanol solution (1.2 mL). The sample was refrigerated at 4 °C for a night and subjected to vortexing for 30 s every 30 min, for a total of six times, to ensure efficient extraction. Subsequently, it was centrifuged at 12,000 rpm for 10 min, and the extracts were filtered using a 0.22 μm pore size prior to UPLC-MS/MS analysis. Three biological replicates were conducted for each treatment. Quality control (QC) was carried out by mixing potato sample extracts and was repeated three times.

### 2.4. Preparation and Extraction of Anthocyanin, Flavonoid, Carotenoid, and SGA

The samples of potato tuber were subjected to freeze-drying, followed by grinding into a powder (30 Hz, 1.5 min) and subsequent storage at −80 °C until the analysis. (1) Anthocyanins: A sum of 50 mg of powder was measured and extracted using 0.5 mL of a methanol/water/hydrochloric acid solution (500:500:1, *v*/*v*/*v*). The mixture was vigorously mixed for a span of 5 min and then sonicated for an additional 5 min. Subsequently, it was centrifuged at a speed of 12,000 rpm at a temperature of 4 °C for a duration of 3 min. The remaining solid was re-extracted by repeating the aforementioned process under the same conditions. (2) Flavonoids: A sum of 20 mg of powder was measured and extracted using 0.5 mL of 70% methanol. Five microliters of internal standards (4000 nmol/L) was incorporated into the extract as internal standards for quantification. The extract underwent sonication for half an hour and was centrifuged at 12,000 rpm at 4 °C for a span of 5 min. (3) Carotenoids: A sum of 50 mg of powder was measured and extracted using 0.5 mL of a blend of n-hexane/acetone/ethanol (1:1:1, *v*/*v*/*v*), and 10 μL of a mixed internal standard solution (20 μg/mL) was added to the extract as a means of internal standardization for quantification. The extract was subjected to vortexing for a duration of 20 min at ambient temperature. The supernatants were isolated following centrifugation at 12,000 rpm for 5 min at 4 °C. The remaining residue was re-extracted by reiterating the aforementioned steps under the same conditions, which was then evaporated to complete dryness and reconstituted in a blend of MeOH/MTBE (1:1, *v*/*v*).

SGAs: The potato tubers were cut into cubes measuring approximately 1 mm × 1 mm × 1 mm. Post-treatment, a fresh sample weighing approximately 1 g was placed in a 10 mL centrifuge tube, to which 10 mL of a 10% acetic acid aqueous solution was added. The extraction of SAG was carried out in accordance with our previous research [16].

The collected supernatants were filtered utilizing a membrane filter (0.22 μm, hanghai Anpu Experimental Technology Co., Ltd., Shanghai, China) prior to undergoing LC-MS/MS analysis for all samples.

### 2.5. UPLC-MS/MS Parameters

The extracts were subjected to a widely targeted metabolomics analysis by employing a UPLC-ESI-MS/MS system (UPLC, SHIMADZU Nexera X2; MS, Applied Biosystems 4500 Q TRAP, Applied Biosystems, Waltham, MA, USA). The analytical parameters included a column of AgilentSB-C18 (100 × 2.1 mm, 1.8 µm) and a mobile phase consisting of solvent A (pure water with 0.1% formic acid) and solvent B (acetonitrile with 0.1% formic acid). The gradient program was structured as follows: 95% A and 5% B at 0 min; gradual changing to 5% A and 95% B from 0 to 9 min, lasting for 1 min; and finally, reducing back to 95% A and 5% B from 10 to 11.1 min, held for 2.9 min. The flow rate was set at 0.35 mL/min, the column oven was set at 40 °C, and the injection volume was set at 4 μL.

A Turbo Ion-Spray electrospray ionization (ESI) interface-equipped triple quadrupole linear ion trap mass spectrometer (Q TRAP), namely the AB4500 Q TRAP UPLC/MS/MS System, was employed to conduct linear ion trap (LIT) and triple quadrupole (QQQ) scans. The device was operated under the control of Analyst 1.6.3 software (AB Sciex) in both positive and negative ion modes. The ESI source parameters were configured as follows: ion source temperature of 550 °C, ion spray voltages of 5500 V (for positive ion mode) and −4500 V (for negative ion mode), gas pressures of I and II, and curtain gas levels at 50, 60, and 25 psi, respectively. The instrument was calibrated using 10 and 100 μmol/L polypropylene glycol solutions in QQQ and LIT modes, respectively, for tuning and mass calibration. QQQ scans were executed as multiple reaction monitoring (MRM) experiments with collision gas (nitrogen) set to medium.

The quantities of anthocyanins, flavonoids, and carotenoids within the sample extracts were determined by means of a UPLC-ESI-MS/MS device (UPLC, ExionLC™ AD; MS, Applied Biosystems 6500 Triple Quadrupole, AB SCIEX, Marsiling Industrial Zone, Singapore). (1) The method for analyzing anthocyanins involved the following parameters: the column used was WatersACQUITY BEH C18 (100 × 2.1 mm, 1.7 µm); the solvent system consisted of water (0.1% formic acid) and methanol (0.1% formic acid). The gradient program was structured as follows: 95% A and 5% B at 0 min; gradual changing to 50% A and 50% B from 0 to 6 min; gradual changing to 5% A and 95% B from 6 to 12 min, lasting for 2 min; and finally, reducing back to 95% A and 5% B at minutes, held for 2 min. The flow rate was 0.35 mL/min; the temperature was set at 40 °C; and the injection volume was 2 μL. (2) The analytical parameters for flavonoids were as follows: A Waters ACQUITY UPLC HSS T3 C18 column with a size of 100 × 2.1 mm and a particle size of 1.8 µm was used. The solvent system consisted of water containing 0.05% formic acid (A) and acetonitrile with 0.05% formic acid (B). The gradient program was structured as follows: 90% A and 10% B at 0 min, gradual changing to 80% A and 20% B from 0 to 1 min, gradual changing to 30% A and 70% B from 1 to 9 min, gradual changing to 5% A and 95% B from 9 to 12.5 min, gradual changing to 5% A and 95% B from 12.5 to 13.5 min, and gradual changing to 90% A and 10% B from 13.5 to 15 min. The flow rate was established at 0.35 mL/min, and the temperature was maintained at 40 °C. The injected volume amounted to 2 μL. (3) The specifications for carotenoid analysis were as follows: The column used was a YMC C30 (100 × 2.0 mm, 3 μm). The solvent blend consisted of methanol/acetonitrile (1:3, *v*/*v*) containing 0.01% BHT and 0.1% formic acid (A), as well as methyl tert-butyl ether with 0.01% BHT (B). The gradient program was structured as follows: 100% A and 0% B from 0 to 3 min, gradual changing to 30% A and 70% B from 3 to 5 min, further gradual changing to 5% A and 95% B from 5 to 9 min, and finally, reducing back to 100% A and 0% B from 10 to 11 min. The flow rate was maintained at 0.8 mL/min, and the temperature was set at 28 °C. The injected volume was 2 μL.

Triple quadrupole linear ion trap mass spectrometers (QTRAP) with ESI Turbo Ion-Spray or APCI Heated Nebulizer interfaces were utilized for LIT and QQQ scans. These instruments operated in either positive ion mode (for anthocyanins and carotenoids) or in both positive and negative ion mode (for flavonoids), and they were controlled using Analyst 1.6.3 software (Sciex). (1) The operation parameters for ESI source of anthocyanins were as follows: positive ion source, 550 °C source temperature, 5500 V ESI voltage, and 35 psi curtain gas. (2) The operation parameters for ESI source of flavonoids were as follows: the ion source was ESI+/−; the source temperature was set at 550 °C; the ESI voltage stood at 5500 V (positive) and −4500 V (negative); and the curtain gas pressure was set at 35 psi. (3) The operation parameters for APCI source of carotenoids were as follows: ion source was APCI+, source temperature was set at 350 °C, and the curtain gas pressure was 25 psi. The analysis of the anthocyanin, flavonoid, and carotenoid contents was conducted using the multiple reaction monitoring (MRM) approach.

The concentration of SGA in potato samples was determined, and the instrumental parameters adhered to our established methodological approach [16].

### 2.6. Data on Metabolite Analysis

Utilizing the in-house constructed database, MWDB (Metware Biotechnology Co., Ltd., Wuhan, China), a qualitative assessment of compounds was executed, relying on primary and secondary mass spectrometry data. Throughout the analysis, isotope signatures, as well as repetitive signals, encompassing K^+,^ Na^+^, NH^+^, and repetitive fragment signals of other compounds with elevated molecular weights, were systematically excluded.

The chemicals were analyzed through the application of multiple reaction monitoring (MRM) via triple quadrupole mass spectrometry. The quadrupole initially screened the precursor ions of the target compound, discounting those corresponding to alternative molecular weights. To mitigate the effects of non-target ions, the precursor ions were induced to ionize within the collision chamber and disintegrate into numerous fragment ions, which were subsequently filtered using the triple quadrupole to isolate a distinctive fragment ion. Upon the acquisition of the mass spectrum data for the metabolites, the peak areas of all substances were integrated, while the mass spectrum peaks of the metabolites in the samples were corrected and integrated.

Qualitative parameters encompass the precise mass of metabolites, MS2 fragments, isotopic distribution of MS2 fragments, and retention time (RT). An analysis was conducted to compare the secondary spectrum and RT of the metabolites in the samples with MWDB utilizing Metware Biotechnology Co., Ltd.’s self-developed intelligent secondary spectrum matching technique. Specifically, the MS tolerance and MS2 tolerance were set at 20 ppm, and the RT offset could not exceed 0.2 min. Identification levels are as follows: (1) the correlation values (ranging from 0 to 1) between the MS/MS mass spectra and compounds in MWDB should exceed 0.7; (2) the correlation values (ranging from 0 to 1) between the MS/MS mass spectra and compounds in MWDB should lie between 0.5 and 0.7; and (3) the Q1, Q3, and RT of metabolites should align with those of MWDB. The accurate determination of anthocyanin, flavonoid, carotenoid, and SGA contents was based on relevant information from their respective standards.

Principal component analysis (PCA), hierarchical cluster analysis (HCA), and orthogonal projections to latent structures discriminant analysis (OPLS-DA) were executed using the R programming language’s prcomp function (www.r-project.org (accessed on 31 October 2023)), with the exception that the PCA and HCA were conducted in an unsupervised manner. The dataset was preprocessed with unit variance scaling prior to analysis. The values of variable importance in projection (VIP) for all metabolites were extracted from the OPLS-DA using the first component, and the OPLS-DA score and permutation plots were produced through the R package called MetaboAnalystR (https://www.metaboanalyst.ca/, accessed on 3 December 2023). The data underwent log transformation (log_2_) and mean centering prior to OPLS-DA. In order to avoid overfitting, a permutation test (200 permutations) was executed. Metabolites with notably distinct abundances between groups were recognized by VIP ≥1 and absolute log_2_ (fold change, FC) ≥1.

The metabolites were annotated by referring to the Kyoto Encyclopedia of Genes and Genomes (KEGG) substance database, and the annotated metabolites were subsequently linked to the KEGG pathway database. Subsequently, pathways with metabolites that showed significant regulation were subjected to metabolite set enrichment analysis (MSEA). The significance of these pathways was assessed using the hypergeometric test *p*-values, with a cut-off of *p*-value < 0.05.

The dynamics data of the SGA content were examined through a generalized linear mixed model (GLMM) incorporating log/logit link functions [17,18]. All models incorporated treatment type and tissue as fixed elements, with time introduced as a random variable, weight introduced as a covariant, and SGA concentration serving as the dependent variable. Subsequent comparisons to detect significant influences employed a sequential Bonferroni approach, with Bonferroni adjustments at a significance level of *p* < 0.05. The content of SGA is presented in mg/kg of fresh weight (FW). The aggregate SGA concentration was calculated as the sum of α-chaconine and α-solanine amounts. The data analysis was carried out using SAS v.9.2 (SAS Institute Inc., Cary, NC, USA). The results are displayed as the mean ± SE.

The composition of connected metabolites in the SGA production process was subjected to a one-factor four-level design for hypothesis testing [19]. All models incorporated the treatments as fixed elements, with the aforementioned metabolites being used as the dependent variables. Significant effect comparisons were conducted using the Duncan method with a *p*-value of less than 0.05. Before testing the data for hypotheses, a logarithmic transformation was applied to ensure normal distribution and homogeneity of variance.

## 3. Results

### 3.1. Morphology of Potato Tuber

**Figure 1:**
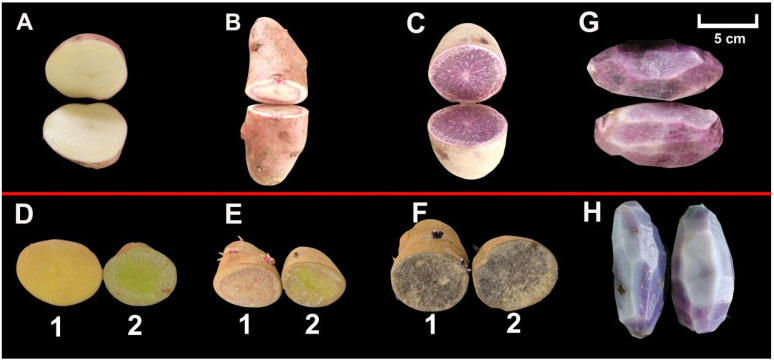
The appearances of potatoes stored in both dark and light environments at 0 and 8 days. Three potatoes are labeled (**A**–**C**) at 0 days. The appearances of injured potatoes stored in dark conditions are referred to as (**D1**–**F1**) at 8 days, while the appearances of injured potatoes stored in light conditions are labeled (**D2**–**F2**) at 8 days. The tuber appearance after steaming is denoted by (**H**), and its CK is denoted by (**G**).

We selected the potato cultivars HZ88 (Figure 1A), SD6 (Figure 1B), and JCH (Figure 1C) as research subjects. The HZ88 flesh is light yellow, the SD6 flesh is colorful, and the JCH flesh is purple. We observed that the appearance of the tuber flesh remained virtually unaltered when stored in dark conditions for 8 days (Figure 1(D1,E1)), yet the other tuber flesh turned green after 8 days of light exposure (Figure 1(D2,E2)). We did not find an appearance difference in the JCH flesh stored under light exposure and dark conditions (Figure 1(F1,F2)). When the potato tubers were steamed, their flesh became lighter in color (Figure 1G,H).

### 3.2. Metabolic Profiling in Potato Tubers

Figure S1A displays a TIC plot derived from a single QC sample, while Figure S1B demonstrates the detection of multiple peaks for metabolites in the MRM mode. The TIC plot portrays the collective intensity of all ions present in the mass spectrum at varying time intervals. A mass spectrum peak depicted in a distinct color signifies a metabolite that has been detected in the multi-peak detection plot. By referring to the local metabolite database, a comprehensive analysis of ion pair data from the potato tuber samples led to the identification of a total of 973 metabolites, encompassing both qualitative and quantitative aspects (Table S1). These compounds encompassed 138 alkaloids, 91 amino acids and derivatives, 155 flavonoids, 26 lignans and coumarins, 148 lipids, 50 nucleotides and derivatives, 67 organic acids, 146 phenolic acids, 6 quinones, 8 steroids, 41 terpenoids, and 97 other metabolites (Table S2), among which 428 were primary metabolites and 545 were secondary metabolites.

### 3.3. Distinguishable Features among the Various Metabolite Profiles

**Figure 2:**
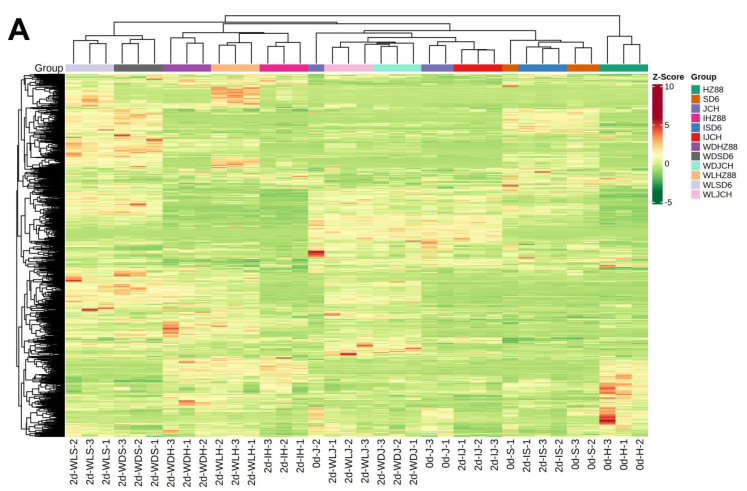

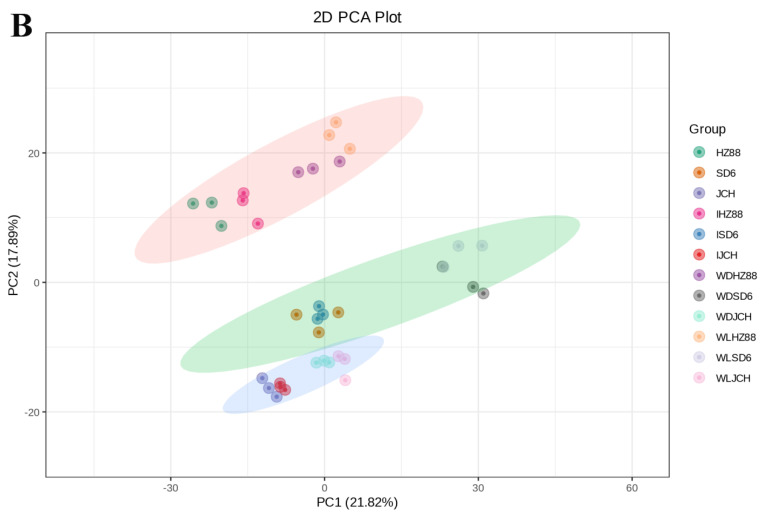
Heatmap (**A**) and PCA plot (**B**) displaying metabolites across various treatments within three potato tubers. (**A**) Each column corresponds to a sample, while each row signifies a metabolite. Abundance levels are denoted by green and red, respectively. (**B**) PC1 and PC2 highlight strong group cohesion and distinct separation among the six potato varieties, respectively; 0 d and 2 d represent storage times of the potato tubers. I, intact potato tuber; W, wounded potato tuber; D and L, potato tubers stored under dark and light conditions, respectively.

Multiple variable statistical analyses were executed to evaluate the variances in the metabolic profiles of various potato tuber treatments. The potato tuber samples were effectively separated in the heatmap (Figure 2A). In the PCA plot, the first two main components (PC1 and PC2) were observed to account for 21.47% and 17.59% of the data’s variance, respectively. The quality control (QC) samples, comprising a blend of potato tuber extracts, were mapped to the same region, suggesting that they possessed similar metabolic profiles and that the sample data were consistent and reproducible. The 39 samples from the four treatments in three potato tubers were categorized into three separate clusters, indicating that each cluster had a slightly different metabolic profile (Figure 2B). Group 1 included the HZ88 accessions with wounding and light exposure treatments (0dHZ88, 2dIHZ88, 2dWDHZ88, and 2dWLHZ88; Figure 2B). Group 2 included the 0dSD6 accessions with wounding and light exposure treatments (0dSD6, 2dISD6, 2dWDSD6, and 2dWLSD6; Figure 2B). Group 3 included the JCH accessions with wounding and light exposure treatments (0dJCH, 2dIHCH, 2dWDJCH, and 2dWLJCH; Figure 2B). It was feasible to distinguish these three clusters from one another quite plainly. In addition, 0dHZ88 and 2dIHZ88 clustered together, and 2dWDHZ88 and 2dWLHZ88 clustered together in the HZ88 tubers. The results of SD6 and JCH were similar to those of HZ88, which indicated that their metabolic profiles were affected by wounding. The PCA findings demonstrated that there were distinctions in the metabolic profiles among different potato cultivars and treatments, with the impact of wounding seemingly surpassing that of light exposure in terms of the metabolic profile alterations (Figure 2B).

In the current study, the variables that contributed to disparities among the seven groups were screened using OPLS-DA. Here, we assessed the disparities between 0dHZ88 and 0dSD6 (R^2^X = 0.64, R^2^Y = 1, Q^2^ = 0.955), between 0dHZ88 and 0dJCH (R^2^X = 0.638, R^2^Y = 1, Q^2^ = 0.951), between 0dJCH and 0dSD6 (R^2^X = 0.595, R^2^Y = 1, Q^2^ = 0.876), between 0dJCH and 2dIJCH (R^2^X = 0.524, R^2^Y = 1, Q^2^ = 0.72), between 0dJCH and 2dWDJCH (R^2^X = 0.52, R^2^Y = 1, Q^2^ = 0.844), between 0dJCH and 2dWLJCH (R^2^X = 0.563, R^2^Y = 1, Q^2^ = 0.891), and between 2dWDJCH and 2dWLJCH (R^2^X = 0.463, R^2^Y = 0.999, Q^2^ = 0.55) via the OPLS-DA mode.

**Figure 3:**
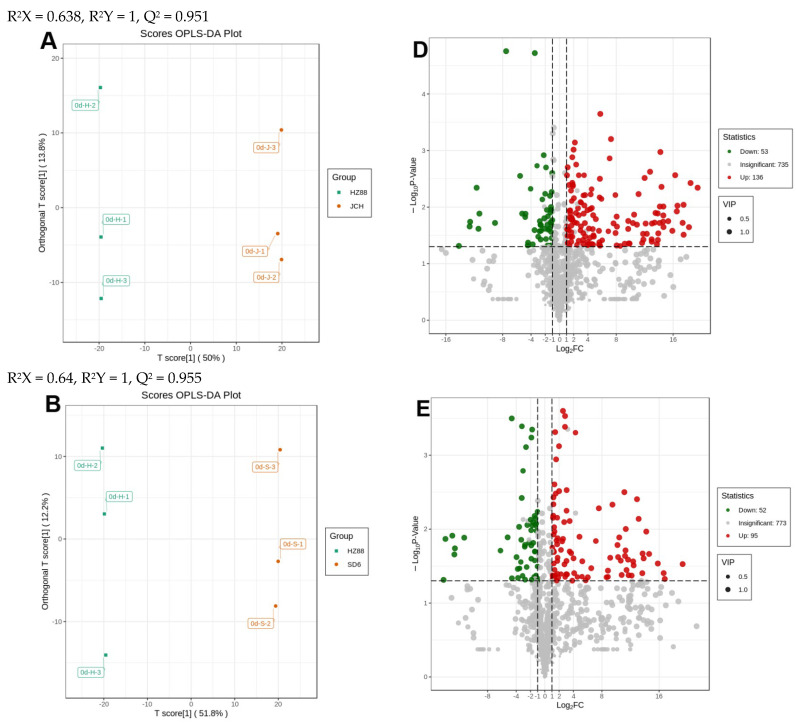

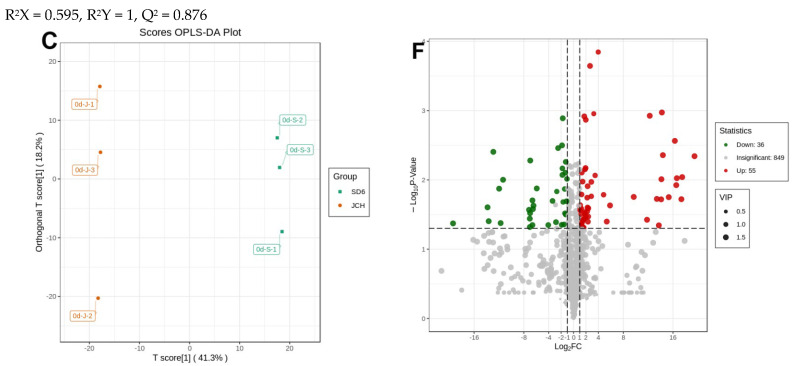
Analysis of metabolomics differences among the three potato flesh types at zero days (0 d). OPLS-DA model graphs (**A**–**C**) and loading plots (**D**–**F**) for HZ88 in comparison to SD and JCH are presented. Volcano plots display the variations in metabolomics expression levels between HZ66, SD6, and JCH. The downregulated differentially expressed metabolites and upregulated differentially expressed metabolites are denoted by green and red spots, respectively; gray spots signify detected metabolites with insignificant differences. Absolute log_2_ (fold change, FC) ≥1, −Log_10_(*p*-value) ≥1.3.

**Figure 4:**
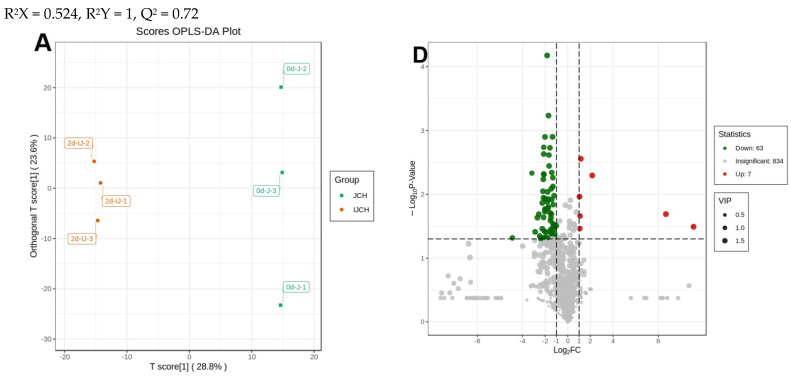

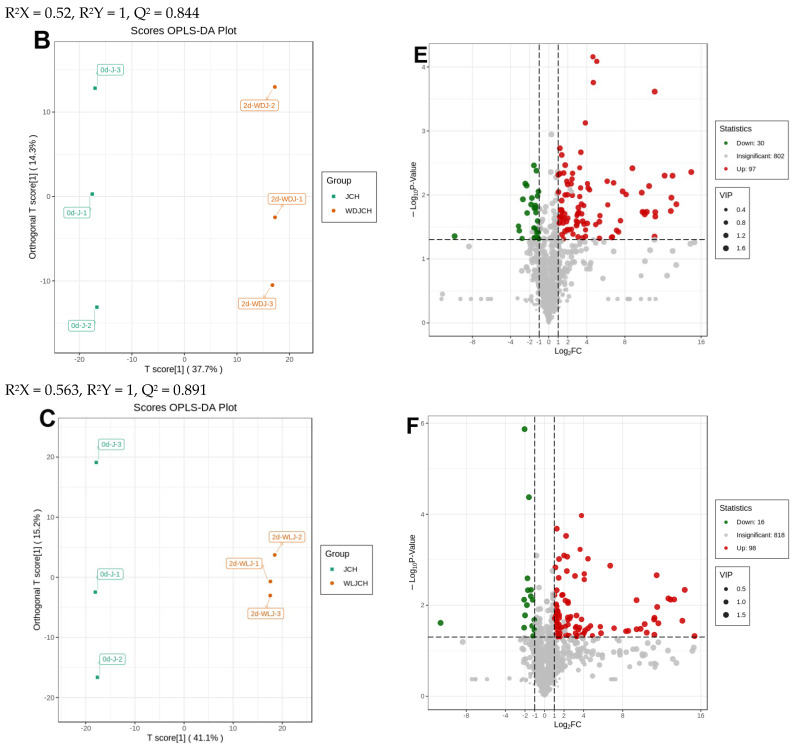
Analysis of metabolomics differences among various treatments in JCH potato tuber flesh. OPLS-DA model graphs (**A**–**C**) and loading graphs (**D**–**F**) of IJCH, WDJCH, and WLJCH (at 2 days) compared to JCH (at 0 days). Volcano plots display the metabolomics expression variations between 0dJCH, 2dIJCH, 2dWDJCH, and 2dWLJCH. The repressed differentially expressed metabolites and activated differentially expressed metabolites are signified by green and red spots, respectively; gray spots denote detected metabolites with insignificant disparities. I, unbroken potato tuber; W, injured potato tuber; D and L, potato tubers stored in darkness and in light conditions, respectively. Absolute log_2_ (fold change, FC) ≥ 1, −Log_10_(*p*-value) ≥ 1.3.

Comparisons between individual elements of the three potato flesh samples, along with the light exposure and wounding treatments in the JCH potato flesh, were executed to identify the metabolites responsible for the noticed discrepancies. Figure 3A–C and Figure 4A–C display OPLS-DA models, where 0dSD6 and 0dJCH are distinct from 0dHZ88, and 2dIJCH, 2dWDJCH, and 2dWLJCH are notably separate from 0dJCH. This suggests significant disparities in the metabolic profiles among the flesh of various potato cultivars and different treatments. Regarding the comparison between 0dJCH and 2dIJCH, as well as between 2dWDJCH and 2dWLJCH, these pairs display a tight clustering in the PCA plots (Figure 2B), yet notable distinctions are detected in the OPLS-DA models (Figure 4A–C), suggesting that the various treatments can be effectively distinguished.

Based on an FC value ranging from ≥2 to ≤0.5 and a VIP value reaching ≥1, a total of 189 different metabolites were identified between 0dHZ88 and 0dJCH (downregulated = 53, upregulated = 136), 147 between 0dHZ88 and 0dSD6 (downregulated = 52, upregulated = 95), and 91 between 0dSD6 and 0dJCH (downregulated = 36, upregulated = 55) (Figure 3D–F). Regarding the light exposure and wounding treatments, a total of 151 distinct metabolites were observed between 0dJCH and 2dIJCH (downregulated = 135, upregulated = 16), 250 between 0dJCH and 2dWDJCH (downregulated = 81, upregulated = 169), 255 between 0dJCH and 2dWLJCH (downregulated = 45, upregulated = 210), 234 between 2dIJCH and 2dWDJCH (downregulated = 14, upregulated = 220), and 292 between 2dIJCH and 2dWLJCH (downregulated = 10, upregulated = 282) (Figure 3D–F).

The list of downregulated and upregulated metabolites for each subgroup is provided in Table S3. The majority of the differential metabolites belonged to the categories of alkaloids, flavonoids, and phenolic acids, with significantly higher concentrations in colored potatoes (0dSD6 and 0dJCH) compared to CK potatoes (0dZH88). The contents of these compounds in the 2dWDJCH and 2dWLJCH treatments were also significantly elevated compared to those of the CK (2dIJCH). The quinones and steroids showed a small change not only in different cultivars, but also in each treatment.

**Figure 5:**
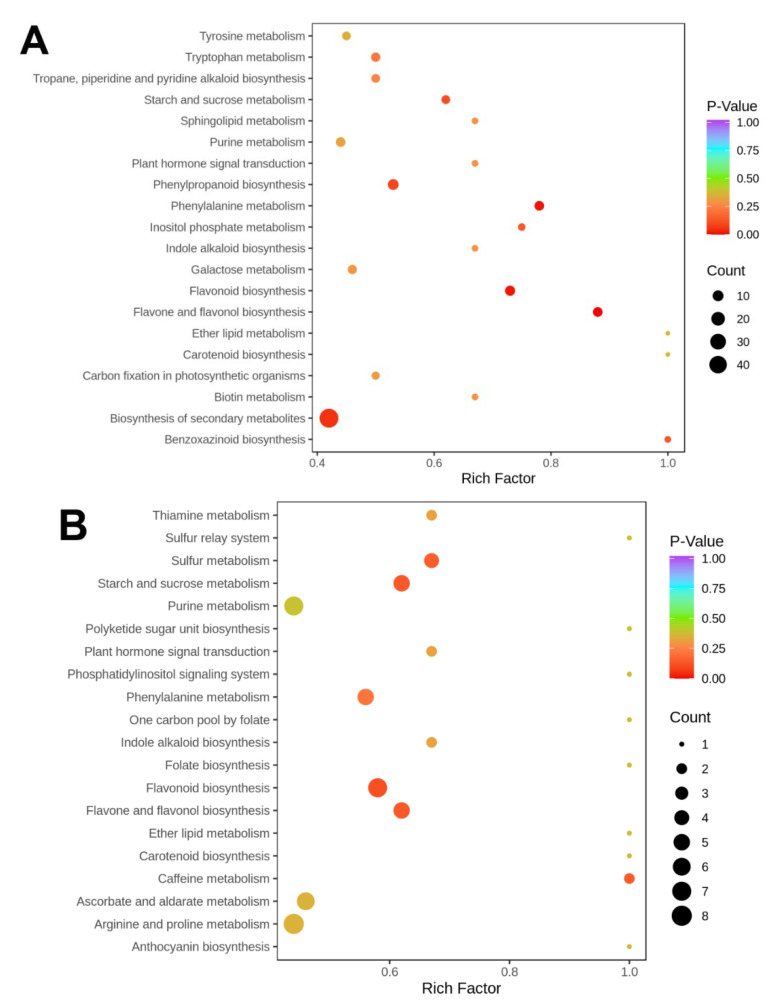

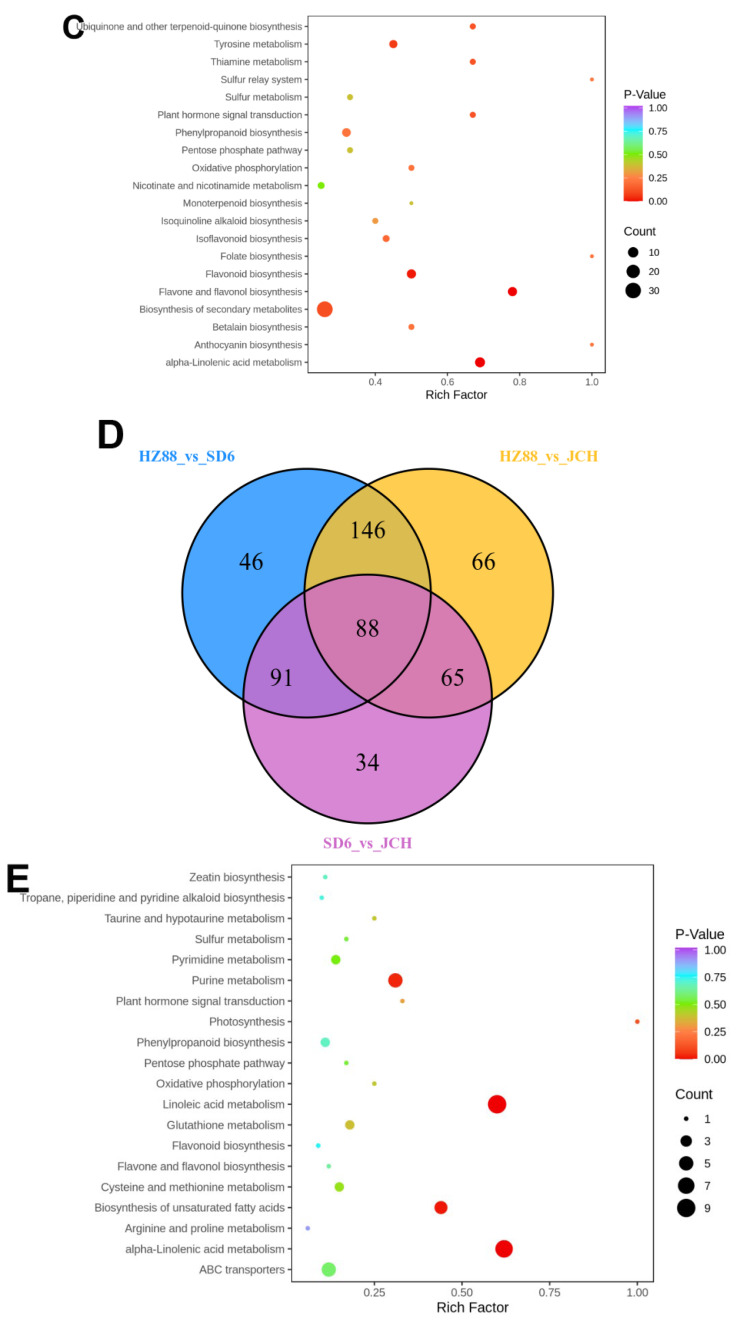

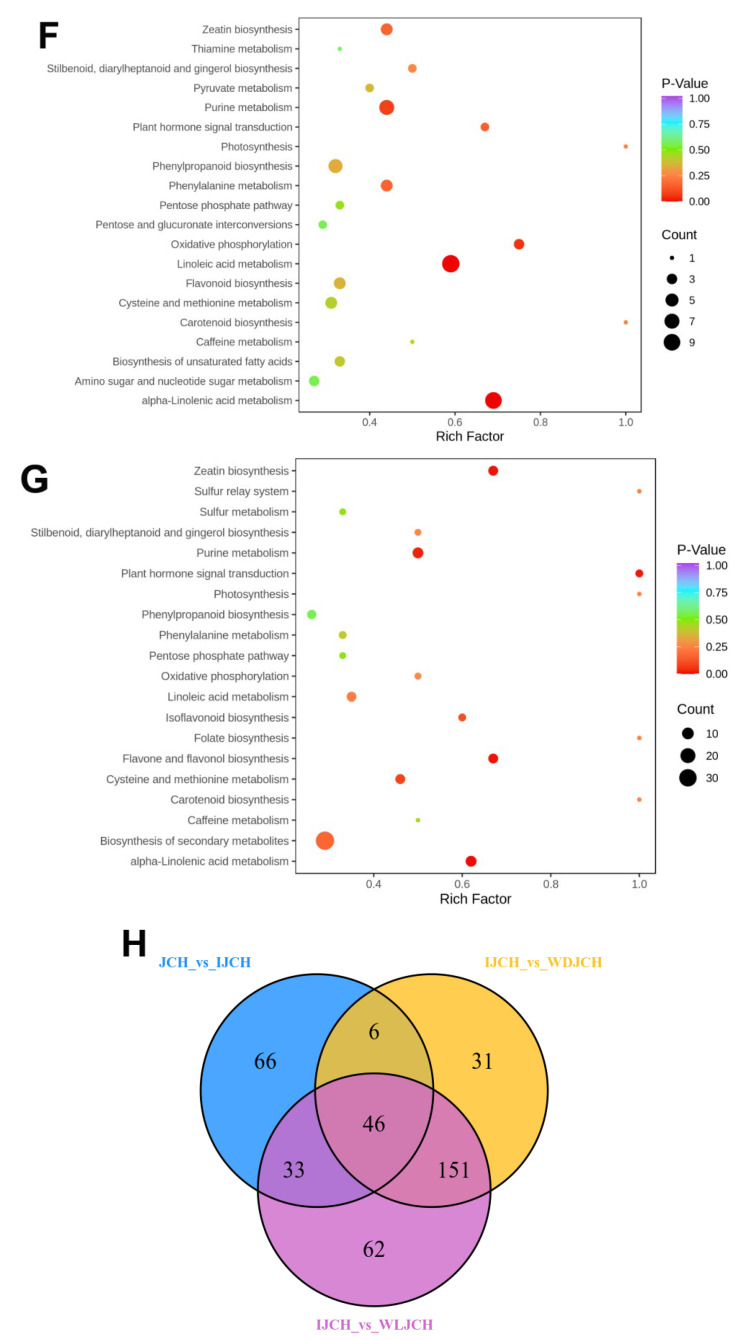
A comparison of metabolite variations between potato varieties (day 0) and treatments (day 2) through Venn diagram and pathway analysis. (**D**,**H**) Venn diagram demonstrating the common and exclusive differentially metabolized compounds within the comparison groups. (**A**–**C**,**E**–**G**) KEGG pathway enrichment analysis based on the metabolite distinctions between the two comparison groups. Each bubble represents a metabolic pathway. The ordinate and bubble size jointly indicate the influencing factors of the pathway. A larger impact factor and the *p*-values of the enrichment analysis are denoted by a larger bubble size and bubble color, respectively. The deeper colors signify greater enrichment levels. I, intact potato tuber; W, injured potato tuber; D and L, potato tubers stored in darkness and light, respectively.

The KEGG database can be used to examine metabolite buildup within a comprehensive network. In the current study, we amplified the varying metabolites of each comparison group and categorized them into diverse pathways. The notably augmented metabolic pathways seen when comparing 0dHZ88 to 0dJCH were associated with “starch and sucrose metabolism”, “phenylpropanoid biosynthesis”, “phenylalanine biosynthesis”, “flavonoid biosynthesis”, “flavone and flavonol biosynthesis”, and “biosynthesis of secondary metabolites” (*p* < 0.05) (Figure 5A). The notably augmented metabolic pathways seen when comparing 0dHZ88 to 0dSD6 were associated with “sulfur metabolism”, “starch and sucrose metabolism”, “flavonoid biosynthesis”, “flavone and flavonol biosynthesis”, and “caffeine metabolism” (*p* < 0.05) (Figure 5B). The pathways associated with “ubiquinone and other terpenoid-quinone biosynthesis”, “tyrosine metabolism”, “thiamine metabolism”, “plant hormone signal transduction”, “flavonoid biosynthesis”, “flavone and flavonol biosynthesis”, and “alpha-linolenic acid metabolism” were notably enriched (*p* < 0.05) in the comparison between 0dSD6 and 0dJCH. (Figure 5C). The data depicted in the Venn diagram demonstrated that a total of 88 metabolites were present in common among the various potato cultivar comparison groups (Figure 5D). The findings implied that the metabolites responsible for the discrepancies exhibited significant variances. To further delve into the distinguishing metabolites of each variety comparison group, the top 10 metabolites with the highest FC values that were upregulated and downregulated in each comparison group were singled out (Figure S2). The upregulated metabolites, in this case, encompassed eight flavonoids and two phenolic acids, while the downregulated metabolites were composed of three alkaloids, one flavonoid, one organic acid, one lignan and coumarin, one terpenoid, and one other metabolite in the comparison of 0dHZ88 vs. 0dJCH (Figure S2A). The upregulated metabolites encompassed eight flavonoids and two phenolic acids, and the downregulated metabolites encompassed three alkaloids, one flavonoid, one lignan and coumarin, one lipid, one organic acid, one phenolic acid, and one terpenoid in the comparison of 0dHZ88 vs. 0dSD6 (Figure S2B). In the comparison of 0dSD6 vs. 0dJCH, the upregulated metabolites encompassed nine flavonoids and one phenolic acid, while the downregulated metabolites were made up of nine flavonoids and one alkaloid (Figure S2C). It is worth noting that the FCs of these compounds were all above 10 in the different cultivar comparison groups.

During the light exposure and wounding treatments, the notably amplified metabolic pathways seen when comparing 0dJCH to 2dIJCH were associated with “purine metabolism”, “linoleic acid metabolism”, “biosynthesis of unsaturated fatty acids”, and “alpha-linolenic acid metabolism” (*p* < 0.05) (Figure 5E). The notably augmented metabolic pathways seen when comparing 0dJCH to 2dWDJCH were associated with “purine metabolism”, “oxidative phosphorylation”, “linoleic acid metabolism”, and “alpha-linolenic acid metabolism” (*p* < 0.05) (Figure 5F). The notably augmented metabolic pathways seen when comparing 0dJCH to 2dWLJCH were associated with “zeatin biosynthesis”, “purine metabolism”, “plant hormone signal transduction”, “isoflavonoid biosynthesis”, “flavone and flavonol biosynthesis”, and “alpha-linolenic acid metabolism” (*p* < 0.05) (Figure 5G). The notably augmented metabolic pathways seen in the comparison between 2dWDJCH and 2dWLJCH were associated with “phenylpropanoid biosynthesis”, “isoflavonoid biosynthesis”, “flavone and flvonol biosynthesis”, and “biosynthesis of secondary metabolites” (*p* < 0.05). The data depicted in the Venn diagram demonstrated that a total of 46 metabolites were common to both the light exposure and wound comparison groups (Figure 5H). These findings indicate that the metabolites responsible for the treatment disparities exhibit significant disparities themselves. The top 10 metabolites with the highest fold change (FC) values in either upregulation or downregulation within each treatment comparison group were singled out (Figure S2). The upregulated metabolites encompassed four alkaloids, three flavonoids, one phenolic acid, one lipid, and one terpenoid. The downregulated metabolites primarily consisted of seven lipids and three flavonoids in the comparison of 0dJCH vs. 2dWDJCH (Figure S2D). In the comparison of 0dJCH vs. 2dWLJCH, the upregulated metabolites were constituted by six flavonoids, two phenolic acids, one organic acid, and one alkaloid, while the downregulated metabolites involved three phenolic acids, two other metabolites, one lipid, one flavonoid, one amino acid and derivative, and one terpenoid (Figure S2E). In the comparison of 2dWDJCH vs. 2dWLJCH, the upregulated metabolites contained five alkaloids, three flavonoids, one phenolic acid, and one lipid, and the downregulated metabolites incorporated six lipids, one alkaloid, one amino acid and derivative, one nucleotide and derivative, and one other metabolite (Figure S2F).

### 3.4. Alkaloid Metabolic Profiling in Potato Tubers

In the current study, a total of 179 different alkaloid metabolites were identified in the five comparative groups; there were 20 markedly different metabolites between 0dJCH and 2dIJCH (upregulated = 5, downregulated = 15), 70 between 2dIJCH and 2dWDJCH (upregulated = 68, downregulated = 2), 68 between 2dIJCH and 2dWLJCH (upregulated = 66, downregulated = 2), and 21 between 2dWDJCH and 2dWLJCH (upregulated = 19, downregulated = 2) (Table S3).

**Figure 6:**
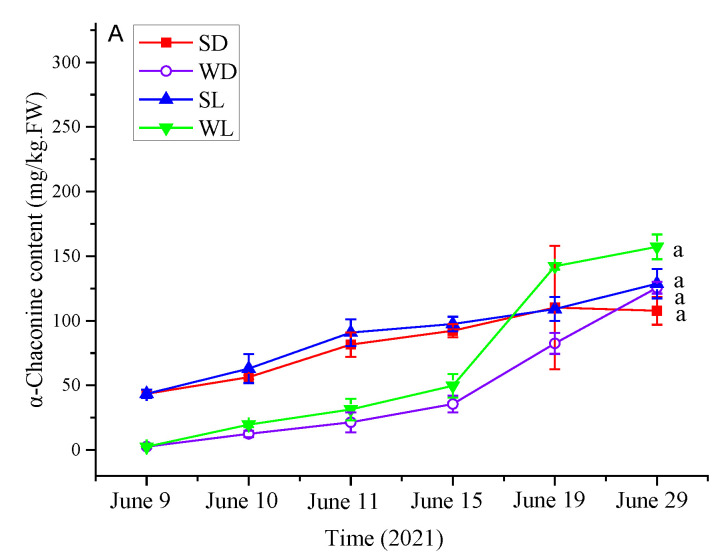

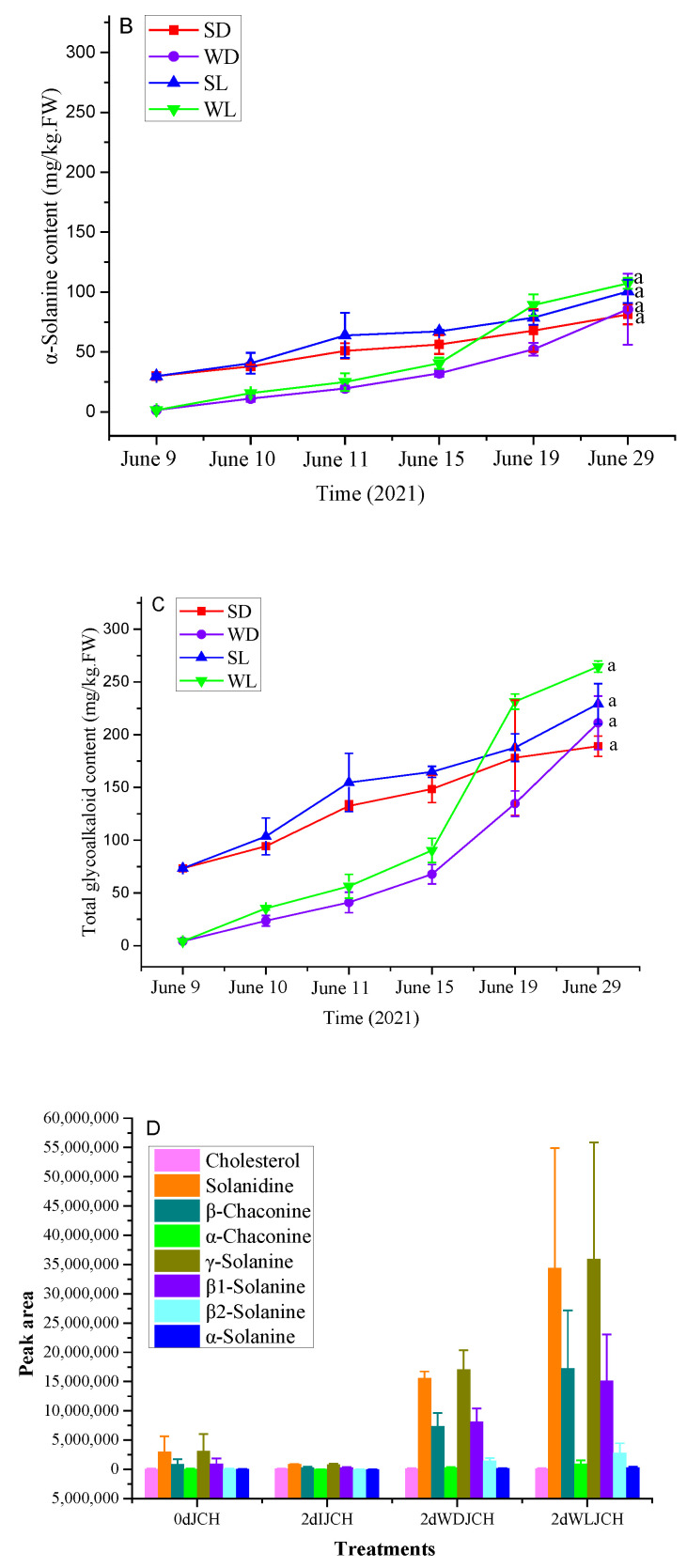
The influence of treatment on SGA metabolite levels in JCH potato tubers. SDJ refers to the wounded tuber skin with dark storage, WD represents the wounded tuber flesh with dark storage, SL represents the wounded tuber skin exposed to light, and WL signifies the flesh of wounded tubers under light exposure. The significant disparities among the groups are denoted by lowercase letter (*p* < 0.05), *n* = 3. (**A**) α-Chaconine, (**B**) α-solanine, (**C**) comprehensive SGA, (**D**) primary metabolites in the SGA synthesis process. I, uninjured potato tuber; W, injured potato tuber; D and L, tubers stored in dark and light conditions, respectively.

We conducted a more in-depth examination of the proportion of key metabolites within the SGA (α- chaconine and α-solanine) synthetic pathway on the second day after storage and found that the metabolite contents (cholesterol: F_(3,8)_ = 0.76, *p* = 0.5458; solanidine: F_(3,8)_ = 6.67, *p* = 0.0144; β-chaconine: F_(3,8)_ = 7.09, *p* = 0.0121; α-chaconine: F_(3,8)_ = 5.76, *p* = 0.0213; γ-solanine: F_(3,8)_ = 7.56, *p* = 0.0101; β1-solanine: F_(3,8)_ = 8.50, *p* = 0.0072; β2-solanine: F_(3,8)_ = 7.36, *p* = 0.0109; α-solanine: F_(3,8)_ = 10.46, *p* = 0.0038) were significantly affected by light exposure and wounding in the SGA metabolic pathway. The solanidine contents in 2dWLJCH and 2dWDJCH were higher than those in 0dJ and 2dIJCH; the β-chaconine content in 2dWLJCH was higher than those in 2dWDJCH, 0dJCH, and 2dIJCH; the α-chaconine content was similar to the β-chaconine content; the γ-solanine, β1-solanine, and β2-solanine contents were similar to the solanidine content; and the α-solanine contents in 2dWLJCH, 2dWDJCH, and 0dJCH were higher than that in 2dIJCH (Figure 6D).

Overall, neither the potato tissue (comprising potato skin and flesh) nor the storage conditions (dark storage and exposure to light) had a significant impact on the SGA levels of the potato tubers (α-chaconine: F_(2,48)_ = 0.69, *p* = 0.5601; α-solanine: F_(2,48)_ = 0.64, *p* = 0.5330; total SGA: F_(2,48)_ = 0.67, *p* = 0.5181) (Figure 6A–C).

### 3.5. The Effect of Cooking Process on Anthocyanin, Carotenoid, and Flavonoid Contents

The target compounds included 108 anthocyanins, 68 carotenoids, and 204 flavonoids in this study. Only six differential anthocyanin metabolites were found, all of which were downregulated compared to the control group (Table S4). The compounds are cyanidin-3-*O*-(6-*O*-p-coumaroyl)-glucoside, cyanidin-3-*O*-galactoside, delphinidin-3-*O*-(6-*O*-p-coumaroyl)-glucoside, delphinidin-3-*O*-galactoside, malvidin-3-*O*-glucoside, and pelargonidin-3-*O*-(6-*O*-p-coumaroyl)-glucoside. Only five differential carotenoid metabolites were found, all of which were downregulated compared to the control group (Table S4). The compounds were β-carotene, zeaxanthin, violaxanthin, neoxanthin, and lutein. Only four differential flavonoids were found, one of which was downregulated (β-mangostin), and three of which were upregulated (jaceosidin, naringenin chalcone, and (-)-gallocatechin) compared to the control group (Table S4).

## 4. Discussion

### 4.1. Metabolites Identified in Potato Tubers

Potato is a vital crop and plays a significant role in ensuring national food security. Potatoes’ appearance, quality, and nutritional value are determined by their skin and flesh color. Thus, three types of potatoes with distinct flesh colors were chosen for metabolic assessment (Figure 1A–C). When the potato tubers were subjected to wounding and light exposure treatments, the potato’s skin and flesh at the wounded site turned green (Figure 1(D2,E2)). No difference in the JCH flesh appearance was observed between the tubers stored under light and dark conditions, which could be because green coloration was not visible in purple flesh (Figure 1(F1,F2)).

In our study, we performed a comprehensive metabolomics examination on three distinct potato cultivars, generating an extensive metabolic profile of potato tubers. Through metabolomics, a total of 973 metabolites were identified through qualitative and quantitative analyses, relying on ion pair data from compounds within potato tubers. The metabolites encompassed 138 alkaloids, 91 amino acids and derivatives, 155 flavonoids, 26 lignans and coumarins, 148 lipids, 50 nucleotides and derivatives, 67 organic acids, 146 phenolic acids, 6 quinones, 8 steroids, 41 terpenoids, and 97 additional metabolites (Table S2). Most of the compounds were alkaloids, flavonoids, and phenolic acids. The minority of the compounds were classified as lignans and coumarins, quinones, and steroids. Most of the differential metabolites were alkaloids, flavonoids, and phenolic acids. However, the quinones and steroids showed a small change not only in different cultivars, but also in each treatment (Table S3). An earlier investigation determined that a total of 505 metabolites were identified, with the majority consisting of lipids, amino acids and their derivatives, phenolic acids, alkaloids, and additional components [14]. The study indicated that the chlorogenic acid in potato peel exhibited promising antioxidant activity against soybean oil oxidation, and its antioxidant effect increased with the increase in the extract dose [20]. Flavonoids, which are potent antioxidant agents, exhibit various biological functions, pharmacological functions, and anticancer and anti-inflammatory properties [21]. For example, naringenin showed anticancer, antioxidant, anti-inflammatory, and antiproliferative activities [22,23]. Kaempferol-3-*O*-glucoside has anti-inflammatory, antioxidant, and protective properties [24]. The antioxidant properties of potatoes are closely related to the components [7,20]. As a result, the current study offers valuable benchmarks for comprehending the defense mechanisms of potatoes against biotic and abiotic stresses, as well as for identifying and isolating the functional components in potatoes.

### 4.2. Metabolite Variations within Cultivars and Treatment Approaches

In the current research, a collective total of 427 distinguishable metabolites were detected in the trio of comparison groups; nevertheless, just 88 metabolites were noticed in the variety comparison groups, indicating that the metabolite profiles of the three potato varieties exhibit a notable disparity. The findings of our research align with the outcomes of other investigations. It was discovered by researchers that potato cultivars have an effect on metabolomics [15]. Friedman et al. found that the contents of the total SGA, phenolic compounds, and flavonoids in potatoes demonstrate a considerable range of variation [7]. Interestingly, “flavonoid biosynthesis” existed in the three comparison groups. In these potato cultivars, colored potato tubers are rich in flavonoids, and the related contents of 85 and 79 flavonoids were higher in 0dSD6 and 0dJCH than that in 0dHZ88, respectively. Various metabolites could potentially account for the hue variation in potato flesh. Flavonoids, in the realm of biomedical and health sciences, serve vital physiological functions. They function as free radical eliminators, antioxidants, and singlet oxygen suppressants [21,25]. Therefore, it is very important to cultivate colorful potato tubers to cater to the diverse demands of consumers for health and nutrition.

The top 10 metabolites with the highest fold changes in upregulation and the top 10 metabolites with the highest fold changes in downregulation for each treatment comparison group were chosen (Figure S2). Most of these compounds were flavonoids (eight upregulated) and alkaloids (three downregulated) in the 0dHZ88 vs. 0dSD6 comparison group, and flavonoids (eight upregulated) and alkaloids (three downregulated) in the 0dHZ88 vs. 0dJCH comparison group; there were nine upregulated flavonoids and nine downregulated flavonoids in the 0dSD6 vs. 0dJCH comparison group. The findings demonstrated that the top 10 highest FCs were noticed in flavonoids and alkaloids. The variation in metabolites among various potato cultivars suggests that the grades of these potatoes differ. Potatoes with relatively high flavonoid contents can be used as natural raw materials for the development and extraction of functional substances, such as natural antioxidants [26].

The previous results indicated that light exposure and wounding increased the SGA levels in potato tubers [27], which might be the result of changes in gene expression [28]. The findings of our study demonstrated that a combination of light exposure and wounding led to a significant increase in the SGA contents within the tubers [12]. As far as we are aware, this is the first study that has been conducted on the impacts of light exposure and wounding on the metabolomics of potato tubers. Throughout the process of potato tuber harvesting and transportation, injuries can occur due to the use of harvesting and relocation equipment [11], and potatoes can be affected by light exposure. Importantly, wound-related and green-related effects caused by light exposure result in substantial food and financial losses. As a result, investigating the metabolic process related to injury and illumination is crucial for minimizing food squander and financial decline in potato bulbs. JCH was chosen for additional exploration.

When potatoes are wounded, the levels of many metabolic compounds increase significantly, which is beneficial for food quality, including beneficial properties such as antioxidant activity. In the current research, a collective total of 395 metabolite differences were detected among the comparison groups (Figure 5H). Only 46 common metabolites were noticed in the comparison groups, implying that the metabolite profiles among the various treatments were significantly distinct. The outcomes from the light exposure and wounding treatments demonstrated that the wounding stress triggered metabolic pathways such as “plant hormone signal transduction” and “flavone and flavonol biosynthesis”, and light affected the synthesis of these substances in these pathways. The top 10 metabolites with the most significant upregulation and the top 10 metabolites with the most significant downregulation with the highest FCs were chosen (Figure S2). Most of these compounds were alkaloids and flavonoids (four and three upregulated, respectively) and lipids and flavonoids (seven and three downregulated, respectively) in the 0dJCH vs. 2dWDJCH comparison group. Most of these compounds were alkaloids and flavonoids (five and three upregulated, respectively) and lipids (six downregulated) in the 0dJCH vs. 2dWLJCH comparison group. Most of these compounds were flavonoids (six upregulated) and phenolic acids (three downregulated) in the 2dWDJCH vs. 2dWLJCH comparison group. The findings suggest that wounding affected alkaloid accumulation and flavonoids are related to light exposure in potato tubers. An earlier investigation proposed that flavonoids executed physiological functions in guarding against biotic or abiotic pressures [5,29]. Consequently, the findings further suggested that the method of safeguarding potatoes from biotic or abiotic stress could potentially be regulated by altering the levels of these metabolites. The decrease in lipids during this period may be related to tuber wound healing [30,31], which needs to confirmed by further research.

### 4.3. Alkaloid Metabolites

In the current study, a total of 179 different alkaloids were identified in the groups; there were 5, 68, 66, and 19 upregulated metabolites in the 0dJCH vs. 2dIJCH, 0dJCH vs. 2dWDJCH, 0dJCH vs. 2dWLJCH, and 2dWDCH vs. 2dWLJCH groups, respectively, and there were 15, 2, 2, and 2 downregulated metabolites in these groups, respectively (Table S3), which suggested that wounding significantly increased the alkaloid contents. The results could be related to the wounded stress in potato tubers [32]. Through a further analysis of the contents of the major compounds in the potato SGA synthetic pathway on the second day after storage, it was found that some metabolic contents, such as that of solanidine, were higher in 2dWDJCH and 2dWLJCH than those in the other treatments. The contents of the other compounds remained in a narrow range in the different treatments, which may be due to the short sampling time (Figure 6). Importantly, the SGA contents within 48 h among the treatments in potato JCH were similar. In our previous work, significant SGA content changes were observed in wounded potato tubers within 48 h [12], which indicated that the accumulation rate of SGA was different in different potatoes after undergoing the same treatment. In the current investigation, the content of SGA in potato skin demonstrated a significant elevation compared to that in the flesh during the initial phase of storage, with a gradual amplification observed throughout the experimental period. However, the SGA contents in the flesh at the wounded site increased sharply from June 15 to 29, 2021, and then remained in a narrow range at the end of storage (Figure 6). Over 80 SGAs have been detected in potato alkaloid [33]. Although the aggregate amounts of α-chaconine and α-solanine constitute approximately 95% of the entire SGA content in potato [34], we further ascertained the absolute quantities of α-chaconine and α-solanine. Importantly, the SGA contents within the 3 mm wound site remained below 7 mg/kg of FW (α-chaconine: 4.09 ± 1.67 mg/kg. FW; α-solanine: 2.46 ± 0.73 mg/kg. FW; total SGA: 6.55 ± 2.21 mg/kg. FW), which is far below the permissible limit of 200 mg/kg of FW.

The effects of SGA poisoning are typified by symptoms such as severe abdominal and gastric pain, inflammation of the intestines, diarrhea, vomiting, fever, rapid heart rate, low blood pressure, and neurological disorders [35], which could be due to a disruption in the membranes and a limitation of acetylcholine esterase functionality [36]. SGA hampers the cell cycle, undermines the structural integrity and permeability of the intestinal mucosal epithelium, restricts cell proliferation, and augments cell programmed demise in small intestinal epithelial cells in mouse [37]. Therefore, it is necessary to breed potato varieties in which SGA accumulation is not sensitive to injury. Given that potato SGA holds properties like antiallergic, antipyretic, anti-inflammatory, hyperglycemic, and antibiotic characteristics [8], potato tubers with relatively high SGA contents can be developed as medicinal raw materials, which can improve the values of potato processing byproducts and reduce waste causing environmental pollution.

### 4.4. The Effect of Cooking on Metabolites

In the current study, a limited quantity of anthocyanin, carotenoid, and flavonoid derivatives (15 out of 271; 5.54%) were significantly changed after steaming, and most of the differential metabolites were downregulated after steaming (11 out of 15, 73.33%). Only three flavonoids (3 out of 15, 20%) were upregulated, which indicated that most metabolites did not change in this cooking process, and they had strong stability. The findings of this research align with the outcomes of other investigations [38], which have suggested that most nutrients and bioactive compounds are partially degraded during cooking [15]. These results may be responsible for the changes in the potato tuber color during cooking (Figure 1G vs. Figure 1H).

## 5. Conclusions

This study assesses the variation in metabolites among different potato cultivars and treatments. A comprehensive analysis of our potato tuber samples resulted in the identification of a total of 971 metabolites. The difference in metabolites among potato cultivars and between treatment (wounding and light exposure) groups changed significantly. The majority of the discriminative metabolites comprised alkaloids, flavonoids, and phenolic acids. Wounding affects alkaloid accumulation, and flavonoids are related to light exposure in potato tubers. In addition, most metabolites (anthocyanin, carotenoids, and flavonoids) did not change during the cooking process. This research makes a significant contribution to understanding the metabolite composition and functional compounds in potato tubers under light exposure and wounding conditions, and it offers theoretical guidance for potato tuber breeding and consumption.

## Data Availability

Data is contained within the article and Supplementary Material.

## Supplementary Materials

The following supporting information can be downloaded at: https://www.mdpi.com/article/10.3390/foods13020308/s1, Figure S1: Total ion current of one quality control sample determined using mass spectrometry detection (A) and multi-peak detection plot of metabolites in the multiple-reaction monitoring mode (B); Figure S2: The top 10 upregulated metabolites and the top 10 downregulated metabolites with the highest fold change values in each comparison group; Table S1: Information of identified compounds; Table S2: Overview of annotated metabolites; Table S3: Metabolites significantly changed in different treatments; Table S4: The effects of steaming on anthocyanin, carotenoid, and flavonoid contents in JCH potato tubers.
