# Supplementary material for: Metabolomics Reveals the Response Mechanisms of Potato Tubers to Light Exposure and Wounding during Storage and Cooking Processes
Source: Foods. 2024 Jan 18;13(2):308. doi: 10.3390/foods13020308 (PMC10814798; doi:10.3390/foods13020308)
Supplement: Supplementary file 1 [file foods-13-00308-s001.zip › foods-2781393-supplementary.pdf]

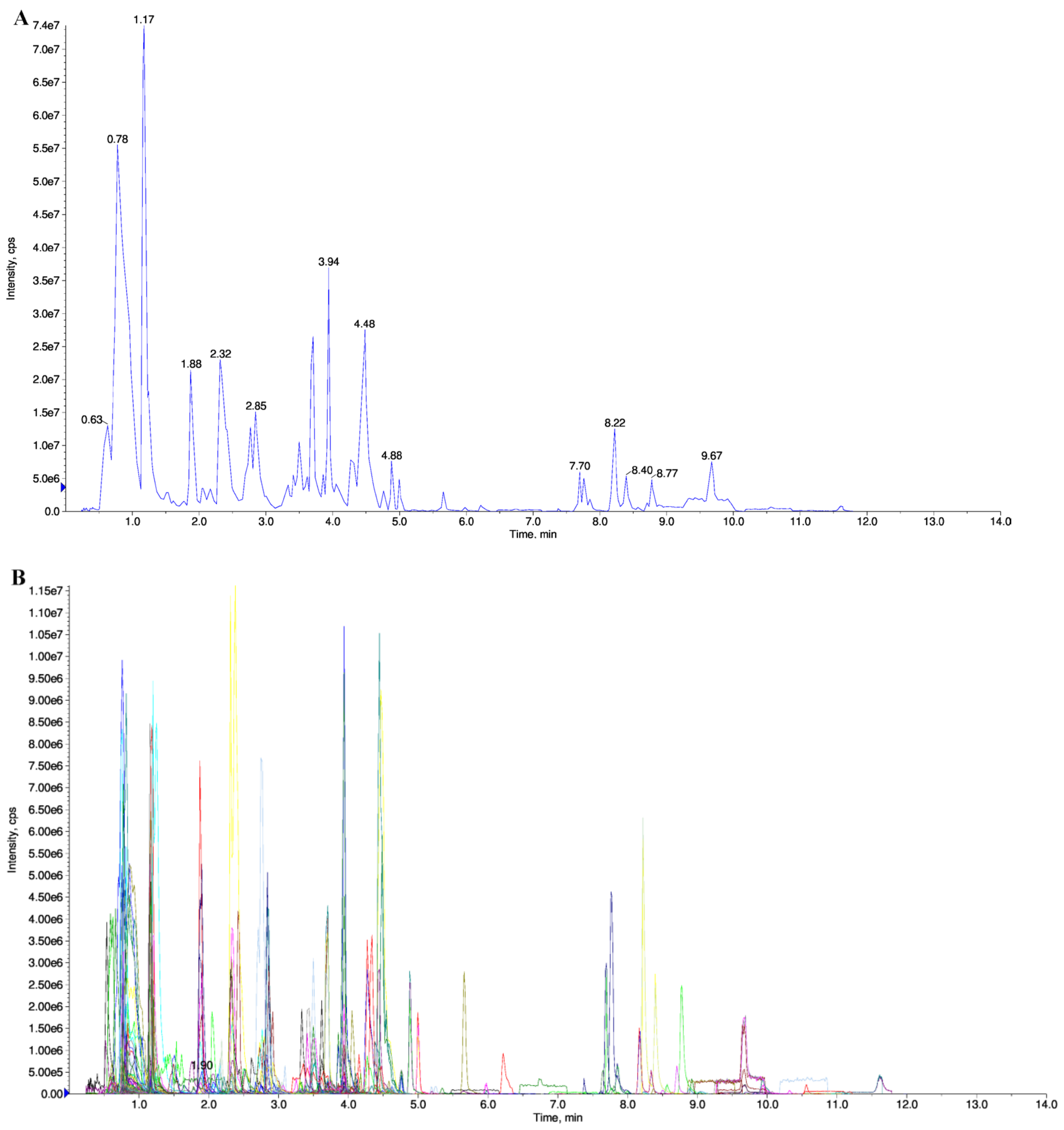

Figure S1. Total ion current of one quality control sample determined using mass spectrometry detection (A) and multi-peak detection plot of metabolites in the multiple-reaction monitoring mode (B).

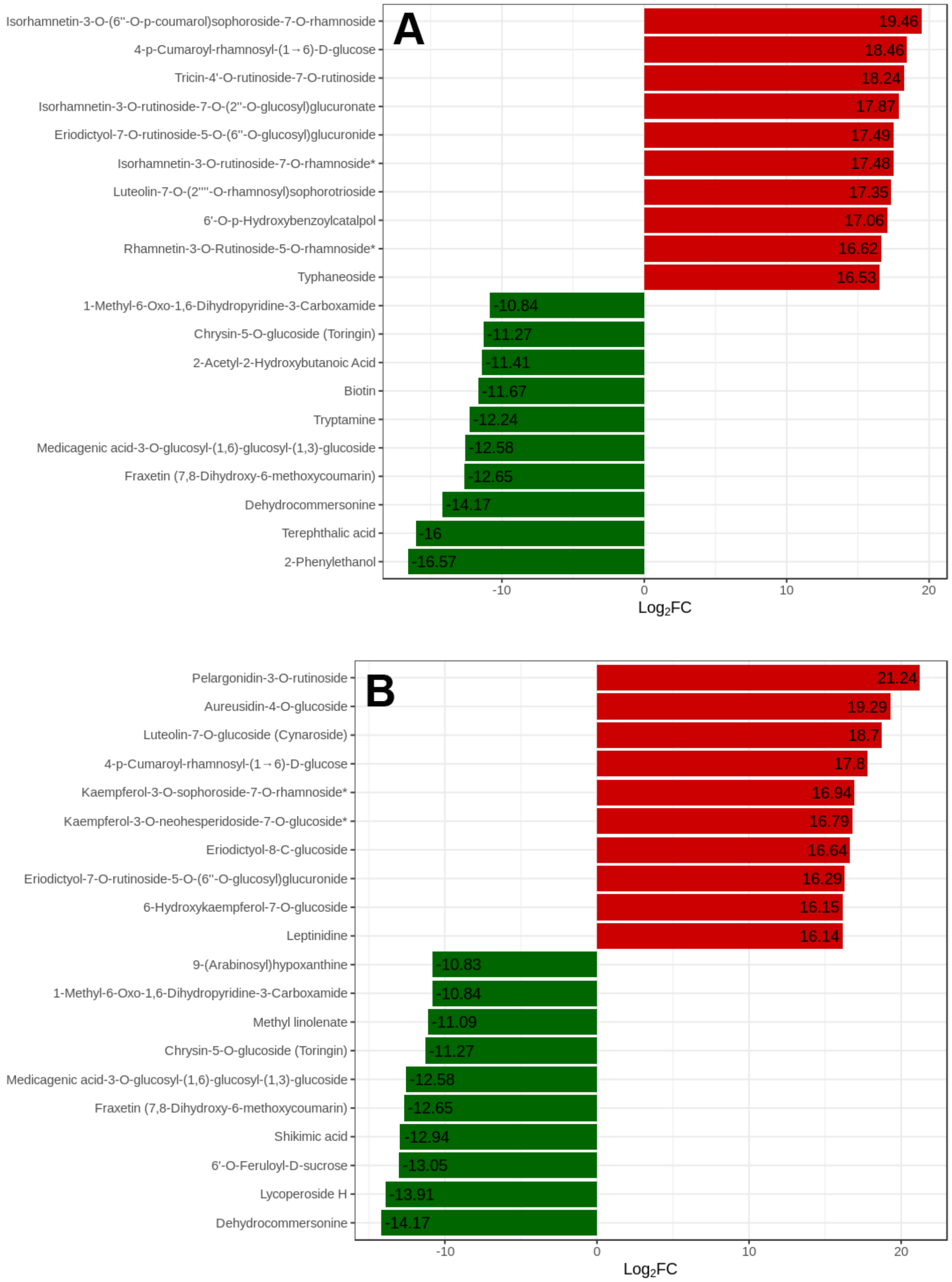
A
Isorhamnetin-3-O-(6''-O-p-coumarol)sophoroside-7-O-rhamnoside
4-p-Cumaroyl-rhamnosyl-(1→6)-D-glucose
Tricin-4'-O-rutinoside-7-O-rutinoside
Isorhamnetin-3-O-rutinoside-7-O-(2''-O-glucosyl)glucuronate
Eriodictyol-7-O-rutinoside-5-O-(6''-O-glucosyl)glucuronide
Isorhamnetin-3-O-rutinoside-7-O-rhamnoside*
Luteolin-7-O-(2''''-O-rhamnosyl)sophorotrioside
6'-O-p-Hydroxybenzoylcatalpol
Rhamnetin-3-O-Rutinoside-5-O-rhamnoside*
Typhaneoside
1-Methyl-6-Oxo-1,6-Dihydropyridine-3-Carboxamide
Chrysin-5-O-glucoside (Toringin)
2-Acetyl-2-Hydroxybutanoic Acid
Biotin
Tryptamine
Medicagenic acid-3-O-glucosyl-(1,6)-glucosyl-(1,3)-glucoside
Fraxetin (7,8-Dihydroxy-6-methoxycoumarin)
Dehydrocommersonine
Terephthalic acid
2-Phenylethanol
19.46
18.46
18.24
17.87
17.49
17.48
17.35
17.06
16.62
16.53
-10.84
-11.27
-11.41
-11.67
-12.24
-12.58
-12.65
-14.17
-16
-16.57
-10
0
10
20
Log2FC
B
Pelargonidin-3-O-rutinoside
Aureusidin-4-O-glucoside
Luteolin-7-O-glucoside (Cynaroside)
4-p-Cumaroyl-rhamnosyl-(1→6)-D-glucose
Kaempferol-3-O-sophoroside-7-O-rhamnoside*
Kaempferol-3-O-neohesperidoside-7-O-glucoside*
Eriodictyol-8-C-glucoside
Eriodictyol-7-O-rutinoside-5-O-(6''-O-glucosyl)glucuronide
6-Hydroxykaempferol-7-O-glucoside
Leptinidine
9-(Arabinosyl)hypoxanthine
1-Methyl-6-Oxo-1,6-Dihydropyridine-3-Carboxamide
Methyl linolenate
Chrysin-5-O-glucoside (Toringin)
Medicagenic acid-3-O-glucosyl-(1,6)-glucosyl-(1,3)-glucoside
Fraxetin (7,8-Dihydroxy-6-methoxycoumarin)
Shikimic acid
6'-O-Feruloyl-D-sucrose
Lycoperoside H
Dehydrocommersonine
21.24
19.29
18.7
17.8
16.94
16.79
16.64
16.29
16.15
16.14
-10.83
-10.84
-11.09
-11.27
-12.58
-12.65
-12.94
-13.05
-13.91
-14.17
-10
0
10
20
Log2FC

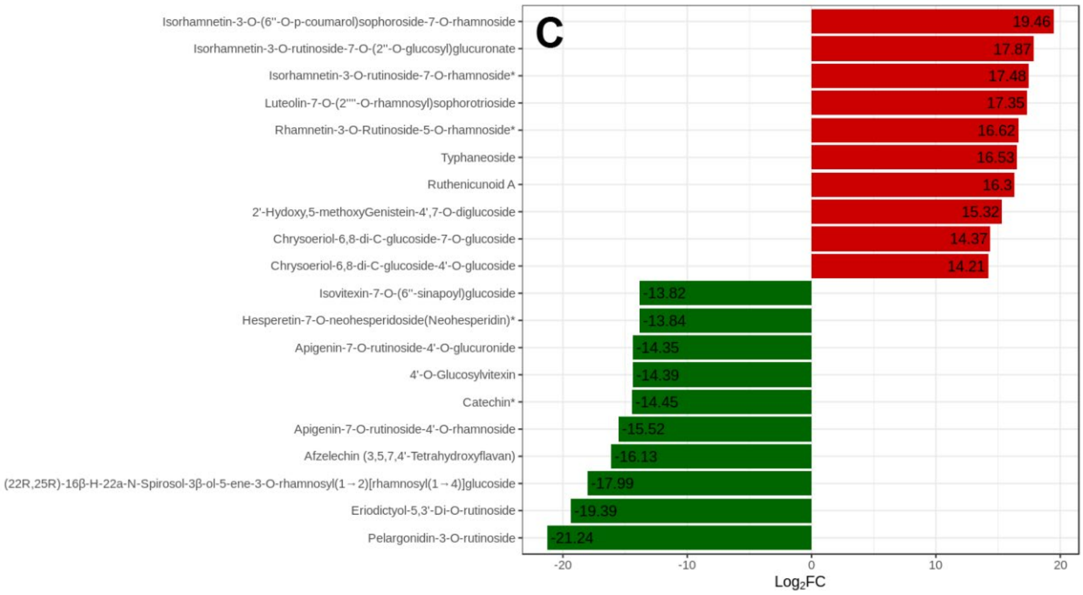
C
Isorhamnetin-3-O-(6''-O-p-coumarol)sophoroside-7-O-rhamnoside
Isorhamnetin-3-O-rutinoside-7-O-(2''-O-glucosyl)glucuronate
Isorhamnetin-3-O-rutinoside-7-O-rhamnoside*
Luteolin-7-O-(2''''-O-rhamnosyl)sophorotrioside
Rhamnetin-3-O-Rutinoside-5-O-rhamnoside*
Typhaneoside
Ruthenicunoid A
2'-Hydoxy,5-methoxyGenistein-4',7-O-diglucoside
Chrysoeriol-6,8-di-C-glucoside-7-O-glucoside
Chrysoeriol-6,8-di-C-glucoside-4'-O-glucoside
Isovitexin-7-O-(6''-sinapoyl)glucoside
Hesperetin-7-O-neohesperidoside(Neohesperidin)*
Apigenin-7-O-rutinoside-4'-O-glucuronide
4'-O-Glucosylvitexin
Catechin*
Apigenin-7-O-rutinoside-4'-O-rhamnoside
Afzelechin (3,5,7,4'-Tetrahydroxyflavan)
(22R,25R)-16β-H-22a-N-Spirosol-3β-ol-5-ene-3-O-rhamnosyl(1→2)[rhamnosyl(1→4)]glucoside
Eriodictyol-5,3'-Di-O-rutinoside
Pelargonidin-3-O-rutinoside
19.46
17.87
17.48
17.35
16.62
16.53
16.3
15.32
14.37
14.21
-13.82
-13.84
-14.35
-14.39
-14.45
-15.52
-16.13
-17.99
-19.39
-21.24
-20
-10
0
10
20
Log2FC

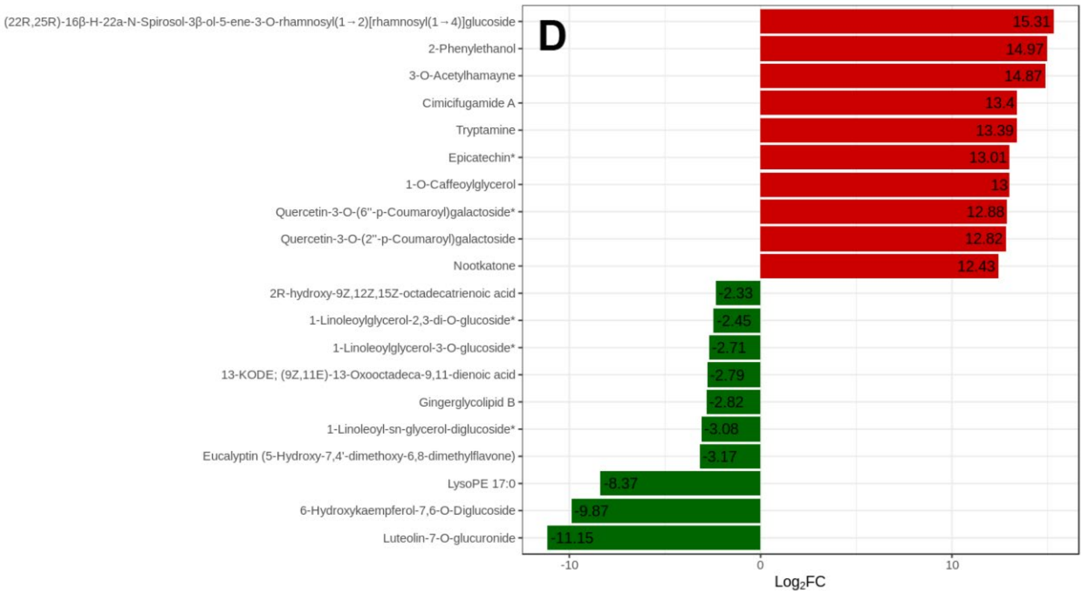
D
(22R,25R)-16β-H-22a-N-Spirosol-3β-ol-5-ene-3-O-rhamnosyl(1→2)[rhamnosyl(1→4)]glucoside
2-Phenylethanol
3-O-Acetylhamayne
Cimicifugamide A
Tryptamine
Epicatechin*
1-O-Caffeoylglycerol
Quercetin-3-O-(6''-p-Coumaroyl)galactoside*
Quercetin-3-O-(2''-p-Coumaroyl)galactoside
Nootkatone
2R-hydroxy-9Z,12Z,15Z-octadecatrienoic acid
1-Linoleoylglycerol-2,3-di-O-glucoside*
1-Linoleoylglycerol-3-O-glucoside*
13-KODE; (9Z,11E)-13-Oxooctadeca-9,11-dienoic acid
Gingerglycolipid B
1-Linoleoyl-sn-glycerol-diglucoside*
Eucalyptin (5-Hydroxy-7,4'-dimethoxy-6,8-dimethylflavone)
LysoPE 17:0
6-Hydroxykaempferol-7,6-O-Diglucoside
Luteolin-7-O-glucuronide
15.31
14.97
14.87
13.4
13.39
13.01
13
12.88
12.82
12.43
-2.33
-2.45
-2.71
-2.79
-2.82
-3.08
-3.17
-8.37
-9.87
-11.15
-10
0
10
Log2FC

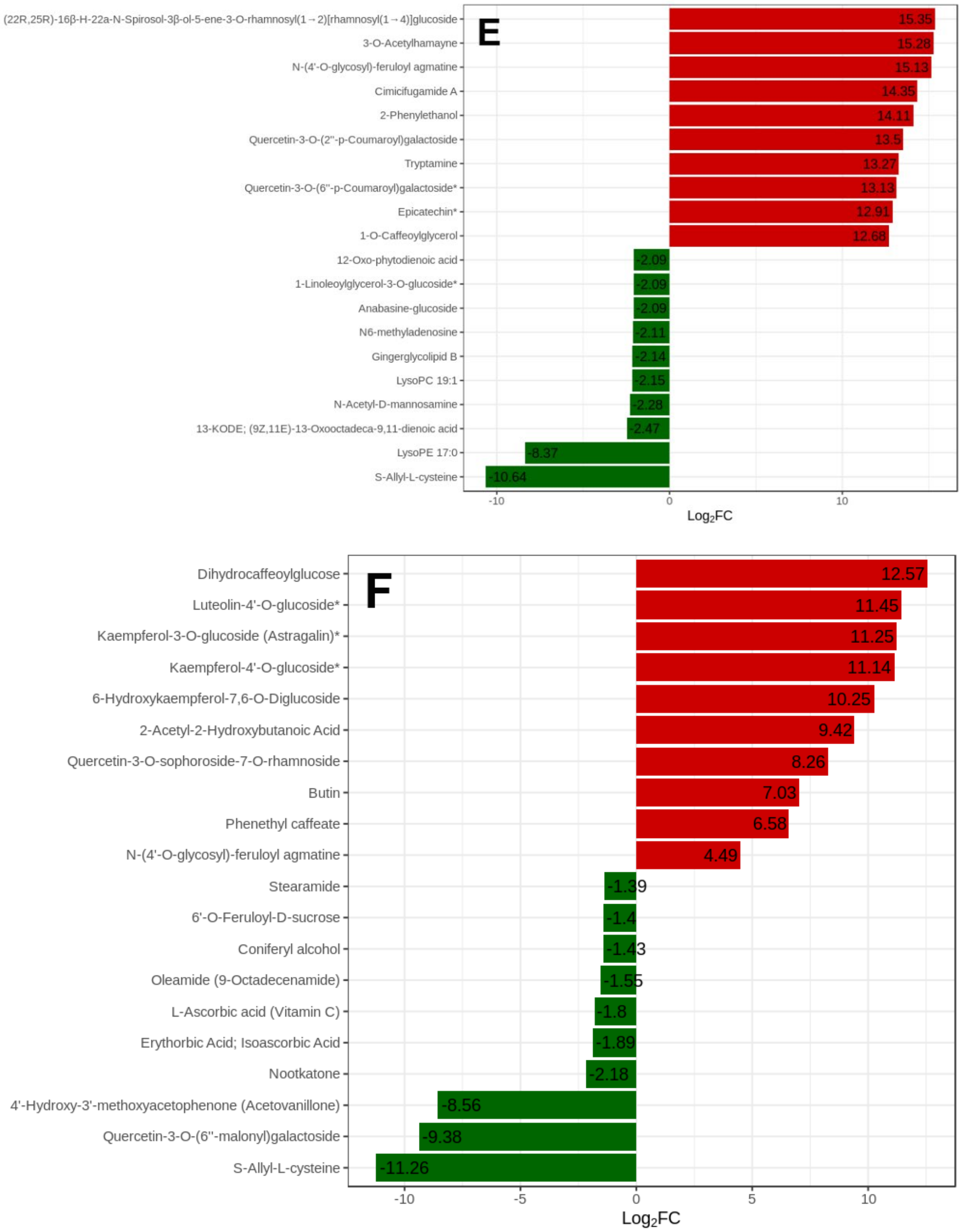

Figure S2. The top 10 upregulated metabolites and the top 10 downregulated metabolites with the highest fold change values in each comparison group. A shows 0dHZ88 vs. 0dJCH, B shows 0dHZ88 vs. 0dSD6, C shows 0dSD6 vs. 0dJCH, D shows 0dJCH vs. 2dWDJCH, E shows 0dJCH vs. 2dWLJCH, and F shows 2dWDJCH vs. 2dWLJCH. Red bar charts are the upregulated metabolites, and green bar charts are the downregulated metabolites. I represents intact potato tuber; W represents wounded potato tuber; and D and L represent potato tubers stored in dark and in light conditions, respectively.

Table S2. Overview of annotated metabolites.

Metabolic type	Number	Percentage (%)
Alkaloids	138	14.18
Amino acids and derivatives	91	9.35
Flavonoids	155	15.93
Lignans and coumarins	26	2.67
Lipids	148	15.21
Nucleotides and derivatives	50	5.14
Organic acids	67	6.89
Phenolic acids	146	15.01
Quinones	6	0.62
Steroids	8	0.82
Terpenoids	41	4.21
Other metabolites	97	9.97

Table S4. The effects of steaming on anthocyanin, carotenoid, and flavonoid contents in JCH potato tubers.

**Name**	**Number of target compounds**	**All significant differences**	**Downregulated compounds**	**Upregulated compounds**
Anthocyanins	108	6	6	0
Carotenoids	68	5	5	0
Flavonoids	204	4	1	3
